# Skeletal Muscle Metastases from Colorectal Adenocarcinoma: A Rare Case Report with Literature Review

**DOI:** 10.3390/reports9020146

**Published:** 2026-05-06

**Authors:** Maria-Mirabela Mihailescu-Marin, Maria-Daniela Chindris

**Affiliations:** 1 Department of Medical and Surgical Specialties, Faculty of Medicine, Transilvania University of Brașov, 500019 Brașov, Romania; 2 Hospice Casa Speranței Foundation, 500074 Brașov, Romania; 3 County Emergency Clinical Hospital, 500326 Brașov, Romania; danielachindris@yahoo.com

**Keywords:** colorectal cancer, skeletal muscle metastasis, MSI-H, oligometastatic disease, PET-CT, case report

## Abstract

**Background and Clinical Significance:** Colorectal cancer (CRC) is the third most common cancer worldwide and the second leading cause of cancer-related death. Skeletal muscle metastases are extremely rare and typically occur in advanced or poorly differentiated tumors. In selected oligometastatic cases, surgical excision can provide symptom relief and requires a multidisciplinary approach. **Case Presentation:** We report a 73-year-old female patient with colonic adenocarcinoma treated with right hemicolectomy and side-to-side mechanical anastomosis, followed by adjuvant CAPOX chemotherapy. The tumor was characterized by MSI-H (microsatellite instability-high) status. During adjuvant treatment (less than 6 months after surgery), she developed progressive right thigh pain, later diagnosed as an intramuscular skeletal muscle metastasis measuring approximately 16 × 13 × 8 cm. The patient underwent en bloc resection of the tumor, followed by adjuvant chemotherapy after metastasectomy. Upon disease progression, first-line chemotherapy in combination with targeted therapy (bevacizumab) was administered. **Conclusions:** Skeletal muscle metastases from colorectal adenocarcinoma are rare. This case emphasizes the importance of recognizing atypical metastatic patterns and suggests that, in selected oligometastatic cases, surgical excision combined with a multidisciplinary approach may improve symptom control and clinical outcomes.

## 1. Introduction and Clinical Significance

Colorectal cancer (CRC) is the third most common cancer worldwide and the second leading cause of cancer-related death, accounting for more than 1.9 million new cases and approximately 904,000 deaths annually. Its incidence is substantially higher in high-income countries compared to transitioning regions and is associated with behavioral and dietary factors, including increased consumption of animal-source foods, a sedentary lifestyle, and a rising prevalence of overweight and obesity [1]. Mortality has declined in many high-income regions due to improved screening programs, early detection, removal of premalignant lesions through colonoscopy, and advances in management and therapy. However, recent increasing trends have been observed among younger adults aged 25–49 years [2].

Approximately 15–30% of patients are diagnosed with metastatic disease, and 20–50% of those with localized disease subsequently develop metastases. The most common metastatic sites include the liver, lungs, peritoneum, and non-regional lymph nodes [3]. In contrast, skeletal muscle metastases are rare, being identified in only 61 of 5170 (1.2%) patients with metastatic cancer in a large computed tomography-based study [4]. The mechanisms underlying the rarity of skeletal muscle metastases are not fully understood but are likely multifactorial. Structural and functional characteristics of skeletal muscle, including its organized architecture, rich vascularization, and constant mechanical activity, may hinder tumor cell adhesion and invasion. In addition, the muscle microenvironment, characterized by metabolic factors such as variations in oxygen tension, lactic acid production, and extracellular matrix stiffness, may be unfavorable for tumor growth. Furthermore, skeletal muscle exhibits enhanced local immune activity and secretes myokines with anti-tumorigenic properties, which may further inhibit cancer cell proliferation and metastatic colonization [5].

We report a rare case of intramuscular skeletal muscle metastasis occurring during adjuvant chemotherapy in a patient with surgically treated colorectal adenocarcinoma. This case highlights an uncommon metastatic presentation, raising diagnostic challenges and therapeutic considerations in an oligometastatic setting, while supporting the role of surgical excision for symptom control within a multidisciplinary approach.

The aim of this report is to describe the clinical presentation, imaging findings, therapeutic management, and prognostic implications of this rare metastatic site, underscoring the importance of multidisciplinary evaluation and a personalized treatment strategy in atypical tumor progression.

## 2. Case Presentation

A 73-year-old female with a history of surgically treated poorly differentiated adenocarcinoma of the ascending colon, complicated by a secondary lesion of the right thigh that was surgically excised, previously treated with chemotherapy and palliative radiotherapy for progressive disease, and presenting with severe pain syndrome, was admitted to an inpatient hospice care unit for symptom control, supportive care, and family respite. The disease was initially classified as stage IIIB resected right colon adenocarcinoma. During adjuvant therapy, the patient developed a solitary distant metastasis, consistent with oligometastatic disease progression.

Her past medical history included well-controlled grade 2 arterial hypertension, type 2 diabetes mellitus managed with oral antidiabetic agents, and previous surgical history of total hysterectomy with bilateral adnexectomy. There was no significant family history of malignancy. Regarding toxic exposures, the patient was a heavy smoker with an estimated smoking history of approximately 50 pack-years.

The oncological history revealed an initial presentation with altered bowel habits predominantly characterized by constipation, unintentional weight loss of approximately 10 kg over a three-month period, and anemia. A colonoscopic examination performed in early 2023 identified a dolichocolon with multiple loops and a proliferative, exophytic tumor causing luminal stenosis at the level of the hepatic flexure. Histopathological analysis of the colonic tumor biopsy confirmed a poorly differentiated, high-grade (G3), infiltrative adenocarcinoma.

Initial staging investigations showed no evidence of distant metastatic disease. The first therapeutic intervention consisted of a right hemicolectomy with mechanical side-to-side anastomosis, performed shortly thereafter. Histopathological examination of the surgical specimen revealed a poorly differentiated (G3) colonic adenocarcinoma with tubular formation < 50%, staged as pT3N1a (1 of 11 lymph nodes involved), with vascular and lymphatic invasion present and negative resection margins (R0). Immunohistochemical analysis demonstrated a Ki-67 proliferation index of 75–80%, consistent with high mitotic activity.

Based on the histopathological findings and stage IIIB disease, adjuvant chemotherapy was initiated. The patient received an adjuvant CAPOX regimen (capecitabine combined with oxaliplatin), with treatment selection influenced by the patient’s age, slightly over 70 years at the time of surgery.

During adjuvant chemotherapy (cycle 3), the patient reported the onset of pain in the proximal lateral aspect of the right thigh, where a firm, apparently fixed mass measuring approximately 4 cm in diameter was palpated. The initially reported 4 cm size corresponded to the clinically palpable portion of the lesion, whereas imaging revealed a substantially larger intramuscular extent. The overlying skin was intact, without erythema or ulceration. No regional lymphadenopathy was detected. Neurovascular examination of the limb was unremarkable, with preserved motor and sensory function and palpable distal pulses.

A hip radiograph showed no evidence of bone metastases. Further evaluation with contrast-enhanced CT of the thorax, abdomen, and pelvis (CT TAP) demonstrated postoperative changes consistent with right hemicolectomy and a mechanical side-to-side anastomosis, without evidence of locoregional recurrence or pulmonary, abdominal, or nodal metastases. However, a previously unreported intramuscular lesion involving the right gluteus medius muscle was identified.

**Figure 1:**
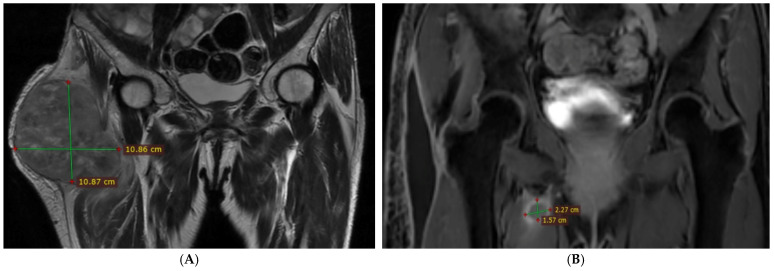
Contrast-enhanced pelvic MRI (coronal plane). (**A**) Preoperative image demonstrating a tumoral mass located in the lateral and anterolateral aspect of the right thigh. (**B**) Postoperative image showing tumor recurrence within the medial compartment of the right thigh, involving the adductor muscle group, with peripheral contrast enhancement and extension to the adductor brevis muscle.

Subsequent contrast-enhanced pelvic MRI, performed in September 2023, revealed a tumoral mass located in the lateral and anterolateral aspect of the right thigh, subjacent to the iliotibial band and gluteal aponeurosis, with loss of the intervening fat plane. The tensor fasciae latae muscle could no longer be identified, appearing incorporated into the tumor mass. The lesion exerted a mass effect on the vastus lateralis, gluteus medius and minimus, and rectus femoris muscles, with partial obliteration of the demarcation planes, while preserving clear interfaces with the major vascular structures (Figure 1A).

**Figure 2:**
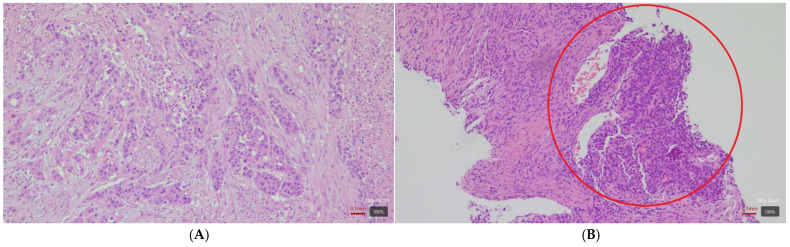
Histopathological features of colonic adenocarcinoma. (**A**) Primary colonic tumor, H&E, ×200, showing infiltrative growth pattern with high-grade tumor budding (>8 buds). (**B**) Metastatic lesion, H&E, ×200, displaying clusters and cords of neoplastic cells with hyperchromatic, elongated (spindle-shaped), pleomorphic nuclei, with morphological features similar to the primary tumor.

A biopsy of the intramuscular lesion was performed. Histopathological and immunohistochemical findings consistent with a poorly differentiated carcinoma of gastrointestinal (colorectal) origin, showing morphological features similar to those of the primary tumor (Figure 2A,B).

Further staging with PET-CT confirmed a metabolically active intramuscular lesion in the right thigh, without evidence of additional pathological uptake.

In October 2023, the patient underwent en bloc surgical resection of the tumor. The excised mass measured 16 × 13 × 8 cm. Histopathological and immunohistochemical analysis confirmed a poorly differentiated metastatic carcinoma of colorectal origin, with negative resection margins.

Considering the PET-CT findings, consistent with oligometastatic disease (a single intramuscular metastasis of the right thigh), the decision for surgical resection was based on the solitary nature of the lesion and the absence of other metastatic sites. Following en bloc resection with negative margins, adjuvant CAPOX chemotherapy was continued after metastasectomy, whereas adjuvant radiotherapy was not indicated due to the R0 resection.

Postoperative MRI performed in early November 2023 revealed a lesion within the right adductor minimus muscle, associated with edema and local mass effect, extending to the adductor brevis muscle (Figure 1B).

Given its imaging characteristics, which differed from the previously excised metastasis, and the presence of predominantly central diffusion restriction, the lesion raised suspicion for an intramuscular abscess, requiring careful differential diagnosis from a possible secondary metastatic lesion.

In this context, adjuvant chemotherapy was continued, with a total of five cycles of the CAPOX regimen administered through March 2024.

Imaging assessment in mid-March 2024 demonstrated significant dimensional progression of the right adductor minimus lesion, increasing from 3.1 × 3.5 × 3.6 cm to 5.2 × 5.2 × 6.5 cm. The mass exhibited mildly lobulated contours and was relatively well defined by a peripheral pseudocapsule. In addition, right external iliac lymphadenopathy measuring up to 2.7 × 1.0 cm was identified.

Due to progressive disease, first-line palliative chemotherapy with the FOLFIRI regimen (including irinotecan) was initiated. Considering the patient’s and family’s preference to seek further orthopedic consultation regarding potential reintervention, the addition of the monoclonal antibody bevacizumab was deferred due to the increased risk of bleeding and delayed wound healing.

After one cycle of FOLFIRI in April 2024, chemotherapy was temporarily interrupted for approximately two months while additional investigations were performed and surgical consultations were obtained. The surgical intervention and a significant proportion of subsequent imaging were carried out at a different university center, resulting in delays in therapeutic decision-making and limiting a coordinated multidisciplinary team (tumor board) approach.

The patient underwent a contrast-enhanced CT TAP in June 2024, which revealed a heterogeneous mass in the uncinate process of the pancreas measuring 40 × 25 mm. The pancreatic lesion was not biopsied due to the need for prompt resumption of systemic therapy, including chemotherapy and targeted treatment, given the prior treatment interruption and symptomatic progression of the thigh recurrence. In the absence of histological confirmation, the lesion was considered most likely metastatic in the context of systemic disease progression, although a primary pancreatic neoplasm could not be excluded. Additionally, a large, irregular, heterogeneous mass with central necrotic areas measuring 11 × 10 cm (vertical dimension 13 cm) was identified in the right thigh, involving the adductor muscle group, partially encasing the sciatic nerve and the profunda femoris artery, and extending superiorly to the ischiopubic ramus, infiltrating the obturator externus muscle. Given the doubling in size of the thigh mass over three months and its proximity to the superficial femoral artery, the surgical team decided to defer intervention.

Consequently, chemotherapy with FOLFIRI was resumed at the end of June, 2024, in combination with bevacizumab. By September 2024, a total of five cycles of FOLFIRI with bevacizumab had been administered, with limited tolerance, including grade 2 gastrointestinal and hematologic toxicities, resulting in temporary treatment delays and dose adjustments.

At this time, the patient presented with severe mixed pain localized to the inner thigh on the right, associated with decreased mobility at the site, incident pain upon movement, and difficulty walking. Due to persistent pain, step 3, analgesic therapy (morphine), was initiated, in combination with a co-analgesic (anticonvulsant). The clinical course was unfavorable, with progressive enlargement of the intramuscular metastasis, development of right inguinal lymphadenopathy, and the appearance of a similar tumor formation on the proximal lateral side of the contralateral thigh. Concurrently, the patient’s performance status deteriorated to an ECOG score of 3, prompting cessation of oncologic treatment.

During a short course in late September 2024, the patient received palliative radiotherapy for analgesic purposes (30 Gy in 10 fractions) to the bilateral thigh lesions, which was well tolerated. Following radiotherapy, the patient reported partial pain relief. Simultaneously, additional testing was performed on the initial colonic tumor specimen. At the time of initial diagnosis, MMR/MSI status had not been assessed, as it was not expected to influence the indication for standard adjuvant chemotherapy according to the guidelines and clinical practice at that time. In the metastatic setting, testing was performed later in the disease course, as access to MSI/MMR evaluation was not yet supported by the national testing program.

Genetic analyses revealed no activating mutations in K- or N-RAS (RAS wild-type), PanTRK immunohistochemistry was negative, and loss of nuclear expression of PMS2 and MLH1 was observed, consistent with high microsatellite instability (MSI-H). Based on these findings, further evaluation for MLH1 promoter hypermethylation and/or BRAF mutation testing was recommended (see Table 1 for chronology of disease progression and associated therapeutic interventions).

Due to severe right thigh pain and deterioration of general condition, the patient was transferred to a hospice facility for palliative care. At the time of transfer, she had an ECOG performance status of 4, a permanent urinary catheter, and moderate abdominal distension. A firm, adherent tumor measuring approximately 15 × 10 cm was noted in the right thigh. Pain was partially controlled with subcutaneous morphine (60 mg/day) in combination with low-dose corticosteroids.

This case report was prepared in accordance with the CARE guidelines.

## 3. Discussion

Colorectal cancer is a malignancy with a high worldwide incidence; however, metastasis to skeletal muscle represents a very rare site of dissemination [6]. The incidence of skeletal muscle metastases (SMM) has been estimated at up to 5.6% among oncology patients in post-mortem series; however, in colorectal cancer it remains extremely low, at approximately 0.028%. A literature review by Kulkarni et al. included 29 studies, corresponding to 30 reported cases between 1970 and the time of analysis. Given that most available data derive from isolated case reports with limited long-term follow-up, firm conclusions regarding mortality remain difficult to establish; however, SMM is generally associated with disseminated disease and poor prognosis [7].

Skeletal muscle is generally considered resistant to metastatic spread due to a combination of mechanical, vascular, metabolic, and immune-related factors that create an unfavorable environment for tumor cell implantation and growth [5]. Age-related alterations in microvascular structure and endothelial function have been described and may lead to changes in tissue perfusion and local microenvironment [8]. However, there is currently no direct evidence that such changes significantly reduce the resistance of skeletal muscle to tumor invasion. These aspects are of particular clinical interest in the context of the rising incidence of colorectal cancer in individuals under 50 years of age [9]. In addition, the muscle microenvironment, characterized by metabolic factors, includes low-molecular-weight, non-protein components such as lactate and pH-related changes, which may further contribute to an unfavorable environment for tumor growth [5].

In patients with colorectal cancer, skeletal muscle metastases may occur through several pathways, including lymphatic or hematogenous spread, or following surgical procedures [10]. The presence of lymphatic and vascular invasion in the primary tumor, combined with the lack of systemic metastatic spread and the solitary intramuscular lesion, favors a lymphatic route of dissemination over a hematogenous mechanism, although a definitive pathway cannot be established.

The literature indicates that muscle metastases usually develop during the course of a previously diagnosed colorectal cancer, while presentation with skeletal muscle involvement as the initial manifestation is extremely rare [10].

The present case is comparable to other reports in the literature and is characterized by a pT3 stage at diagnosis, lymphatic invasion, and, notably, a poorly differentiated tumor associated with microsatellite instability and most likely a BRAF mutation. These features indicate an aggressive biological profile and an unfavorable prognosis.

Skeletal muscle metastases may present with pain, ulceration, or a palpable mass, or may be detected incidentally on imaging studies [7]. However, the most common clinical manifestation remains a painful mass, consistent with the presentation observed in our patient.

To date, research on the imaging characteristics of SMM has been limited. Moreover, this condition is highly heterogeneous, given the variability in primary tumors, metastatic sites, and imaging appearances [11]. Computed tomography is not specific for detecting muscle metastases, whereas magnetic resonance imaging is a valuable tool for lesion characterization and assessment of local extension [12]. Emmering et al. highlighted the usefulness of advanced imaging techniques, particularly fluorodeoxyglucose positron emission tomography (FDG-PET), in the diagnosis of these lesions [13]. FDG-PET can detect metastases that are not visible on contrast-enhanced CT or MRI and has been shown to significantly influence early diagnosis and therapeutic decision-making in more than 50% of cases. Therefore, in the context of suspected SMM, early use of FDG-PET is recommended [7]. Although advanced imaging techniques, particularly FDG-PET, play a key role in the detection of skeletal muscle metastases, definitive diagnosis relies on histopathological confirmation. Ultrasound-guided biopsy represents a widely used approach for musculoskeletal lesions, offering real-time visualization and the advantage of avoiding ionizing radiation. However, CT-guided biopsy may be preferred in selected cases, particularly for deep-seated or poorly visualized lesions, where precise anatomical localization is necessary [14].

The prognosis of patients with skeletal muscle metastases is poor, with reported median survival ranging between 5 and 12 months [10,15]. Overall outcomes are generally unfavorable, a finding supported by a large study on soft tissue metastases reporting a mean survival of 5.4 months from the time of diagnosis [16]. In our case, the patient’s survival from the diagnosis of skeletal muscle metastasis was approximately 15 months, slightly exceeding the median survival reported in the literature (5–12 months). This difference may be explained by the oligometastatic presentation, the absence of widespread visceral metastases at the time of diagnosis, the application of multimodal therapeutic strategies, and the contribution of palliative care in maintaining symptom control and quality of life, although this observation should be interpreted with caution given the limited available data.

The diagnosis of skeletal muscle metastases represents a significant clinical and imaging challenge. Clinical presentation is often nonspecific, and differential diagnosis should primarily include soft tissue sarcomas, muscular infections, and inflammatory lesions [17]. Although CT and MRI provide essential information regarding lesion morphology, PET-CT is particularly useful for assessing both local and distant disease extent. Definitive diagnosis is established by biopsy, which is essential for excluding other etiologies and guiding therapeutic management.

Although skeletal muscle metastases from colorectal cancer have been reported, they remain exceptionally rare. Compared to previously described cases, the present report is notable for the occurrence of a solitary intramuscular metastasis in an oligometastatic setting, allowing for surgical management and symptom control.

To better contextualize our findings, we compared our case with selected representative cases of skeletal muscle metastases from colorectal cancer reported in the literature (Table 2) [7], chosen based on the availability of relevant clinical, therapeutic, and survival data to allow meaningful comparison.

The cases summarized in Table 2 illustrate the heterogeneity of clinical presentation and outcomes, ranging from rapidly progressive disease to prolonged survival in selected cases. In comparison, our case demonstrated a relatively favorable outcome, although this observation should be interpreted with caution given the limited number of reported cases.

Treatment of these metastatic lesions is complex and must be individualized based on disease extent and patient performance status. For isolated metastases, aggressive surgical resection represents the main therapeutic option, with or without preoperative or adjuvant radiotherapy. In the case of disseminated disease, treatment relies on systemic chemotherapy, with therapeutic options expanded by targeted therapies and immunotherapy. These approaches have been shown to improve overall survival and enhance the efficacy of chemotherapy [22,23]. In particular, agents such as nivolumab and pembrolizumab have demonstrated efficacy in patients with metastatic colorectal cancer exhibiting high microsatellite instability and mismatch repair deficiency [24]. In our patient, surgical excision of the thigh tumor was performed, with the treatment considered curative in the setting of oligometastatic disease. Upon progression, palliative chemotherapy and targeted therapy were initiated, as immunotherapy was not reimbursed at that time.

The role of MSI-H/dMMR status in colorectal cancer has become increasingly important, particularly in guiding immunotherapy decisions. MSI-H tumors are characterized by a high mutational burden, which leads to increased neoantigen formation and enhanced immune recognition. This biological profile explains the increased sensitivity of MSI-H colorectal cancers to immune checkpoint inhibitors [25].

Pembrolizumab was approved by the U.S. Food and Drug Administration (FDA) in June 2020 as a first-line treatment for patients with MSI-H/dMMR metastatic colorectal cancer, based on the results of the KEYNOTE-177 trial, which demonstrated significantly longer progression-free survival compared to chemotherapy [26]. More recently, the combination of nivolumab plus ipilimumab received FDA approval in April 2025 following the CheckMate-8HW study [27], further expanding therapeutic options in this setting.

However, in the Romanian context, access to immunotherapy has been limited by reimbursement policies. At the time of this patient’s treatment (2023–2024), national reimbursement for first- or second-line immunotherapy in MSI-H/dMMR metastatic colorectal cancer was not available, which precluded its use. This limitation influenced the management of the case, as certain therapeutic options were not available at the time.

BRAF mutation testing, particularly for the V600E variant, is an important component of the molecular characterization of colorectal cancer, given its prognostic and therapeutic implications [28]. In our patient, this analysis was recommended but not available at the time of writing, representing a limitation in the molecular assessment. The absence of comprehensive molecular profiling (including MSI/MMR and BRAF status at earlier stages) limits a more detailed understanding of the tumor biology and its unusual metastatic pattern, particularly the occurrence of skeletal muscle metastasis.

Although the patient had a significant smoking history, tobacco exposure has been associated with specific molecular subtypes of colorectal cancer, including MSI-high, CIMP-positive, and BRAF-mutated tumors, potentially through epigenetic mechanisms such as DNA methylation. However, these associations remain heterogeneous and not fully understood, and the extent to which smoking-related epigenetic changes directly influence genetic alterations is still under investigation [29].

This case report has several limitations. Its single-patient design and the lack of long-term follow-up data limit the generalizability of the findings, reflecting the scarcity of published data on skeletal muscle metastases in colorectal cancer. In addition, due to the absence of standardized serial imaging measurements, formal response assessment according to RECIST criteria was not applicable.

Further studies are warranted to better understand the pathogenesis of skeletal muscle metastases and to refine diagnostic strategies for early detection, which may facilitate timely multidisciplinary management.

## 4. Conclusions

The present case highlights the rarity of skeletal muscle metastases in colorectal adenocarcinoma and emphasizes the importance of recognizing these unusual clinical manifestations. Such metastases appear more frequently in the context of poorly differentiated tumors and locally advanced disease at diagnosis.

In oligometastatic settings, surgical excision of the lesion may represent a viable option, contributing not only to pain control but also to optimization of therapeutic management. The management of these rare cases requires careful multidisciplinary coordination, underscoring the role of close collaboration between surgery, oncology, and palliative care teams in order to individualize the therapeutic strategy. This case also highlights the importance of early molecular profiling in colorectal cancer, as the delayed identification of MSI-H status may represent a missed opportunity for timely access to immunotherapy.

Clinicians should consider metastatic disease in patients with colorectal cancer presenting with unexplained muscular symptoms, even in the absence of widespread dissemination. Further studies are needed to better define the mechanisms underlying skeletal muscle metastasis and to optimize management strategies in oligometastatic disease.

## Figures and Tables

**Table 1:** Chronology of disease progression and associated therapeutic interventions.

Month/Year	Medical Event	ECOG	Therapeutic Intervention
March 2023	Diagnosis: High-grade, poorly differentiated (G3) infiltrative adenocarcinoma	1	Right hemicolectomy with mechanical side-to-side anastomosis; Adjuvant chemotherapy with capecitabine and oxaliplatin (CAPOX regimen).
September 2023	Skeletal muscle metastasis	2	En bloc resection of the soft tissue tumor located in the anterolateral aspect of the proximal right thigh; Continuation of adjuvant treatment with the CAPOX regimen after metastasectomy
March 2024	Local recurrence involving the adductor minimus muscle	2	Change of chemotherapy line (Palliative chemotherapy with the FOLFIRI regimen)
June 2024	Pancreatic lesion (uncinate process, 40 × 25 mm)	2	Continuation of chemotherapy line (Palliative chemotherapy with the FOLFIRI regimen); bevacizumab
September 2024	Contralateral skeletal muscle metastasis with pain, MSI-H	3	Palliative radiotherapy for pain control
October 2024	Deterioration of performance status	4	Admission to the palliative care unit

**Table 2:** Comparison of selected reported cases of skeletal muscle metastases from colorectal cancer.

Author, Year	Site of Primary Tumor	Stage	Site of Skeletal Metastasis	Timing (Interval in Month)	Treatment	Survival
Tatsuta et al., 2022 [18]	Ascending colon	NR	Cervical (neck muscle)	11	Palliative radiotherapy	2 months
Guo et al., 2021 [19]	Ascending colon	III (pT4N2b cM0)	Right thigh	5	Chemotherapy + bevacizumab	4 months
Yi et al., 2015 [20]	Caecum	NR	Right thenar muscles	Synchronous	Chemotherapy	9 months
Present case	Ascending colon	III (pT3N1 cM0)	Right thigh	6	Surgery + Chemotherapy + bevacizumab + palliative radiotherapy	15 months
Buemi et al., 2019 [21]	Right colon	II (pT3N0 cM0)	Left gluteus muscle	7	Surgery	65 months (disease-free)

Note: Treatment refers to the management of skeletal muscle metastasis. Survival is reported from the time of diagnosis of skeletal muscle metastasis when available. NR = Not reported.

## Data Availability

The original contributions presented in this study are included in the article. Further inquiries can be directed to the corresponding author.
